# A Critical Analysis of Anesthesiology Podcasts: Identifying Determinants of Success

**DOI:** 10.2196/mededu.5950

**Published:** 2016-08-17

**Authors:** Devin Singh, Fahad Alam, Clyde Matava

**Affiliations:** ^1^ Department of Aneshesia and Pain Medicine Hospital for Sick Children University of Toronto Toronto, ON Canada; ^2^ Sunnybrook Health Sciences Centre Department of Anesthesia University of Toronto Toronto, ON Canada; ^3^ eLearning and Technological Innovations Department of Anesthesia University of Toronto Toronto, PE Canada

**Keywords:** anesthesia, podcasts, peer review, success, e-learning, e-resources

## Abstract

**Background:**

Audio and video podcasts have gained popularity in recent years. Increasingly, podcasts are being used in the field of medicine as a tool to disseminate information. This format has multiple advantages including highly accessible creation tools, low distribution costs, and portability for the user. However, despite its ongoing use in medical education, there are no data describing factors associated with the success or quality of podcasts.

**Objective:**

The goal of the study was to assess the landscape of anesthesia podcasts in Canada and develop a methodology for evaluating the quality of the podcast. To achieve our objective, we identified the scope of podcasts in anesthesia specifically, constructed an algorithmic model for measuring success, and identified factors linked to both successful podcasts and a peer-review process.

**Methods:**

Independent reviewers performed a systematic search of anesthesia-related podcasts on iTunes Canada. Data and metrics recorded for each podcast included podcast’s authorship, number posted, podcast series duration, target audience, topics, and social media presence. Descriptive statistics summarized mined data, and univariate analysis was used to identify factors associated with podcast success and a peer-review process.

**Results:**

Twenty-two podcasts related to anesthesia were included in the final analysis. Less than a third (6/22=27%) were still active. The median longevity of the podcasts’ series was just 13 months (interquartile range: 1-39 months). Anesthesiologists were the target audience for 77% of podcast series with clinical topics being most commonly addressed. We defined a novel algorithm for measuring success: Podcast Success Index. Factors associated with a high Podcast Success Index included podcasts targeting fellows (Spearman R=0.434; *P*=.04), inclusion of professional topics (Spearman R=0.456-0.603; *P*=.01-.03), and the use of Twitter as a means of social media (Spearman R=0.453;*P*=.03). In addition, more than two-thirds (16/22=73%) of podcasts demonstrated evidence of peer review with podcasts targeting anesthesiologists most strongly associated with peer-reviewed podcasts (Spearman R=0.886; *P*=.004)

**Conclusions:**

We present the first report on the scope of anesthesia podcasts in Canada. We have developed a novel tool for assessing the success of an anesthesiology podcast series and identified factors linked to this success measure as well as evidence of a peer-review process for a given podcast. To enable advancement in this area of anesthesia e-resources, podcast creators and users should consider factors associated with success when creating podcasts. The lack of these aspects may be associated with the early demise of a podcast series.

## Introduction

Podcasting refers to the distribution of audio or video files in a digital format. These podcasts are viewed on either a user’s personal computer or mobile device, such as a mobile phone. In addition, the use of “really simple syndication” communication protocol to push these audio or video files directly to subscribers is what truly separates podcasts from other means of electronically disseminating information. Podcasting has seen significant growth as a tool in medical education [1-7]. Several studies have concluded that podcasts can be used to enhance a user’s learning experience by providing small, succinct summaries of complex concepts, revision aids, or simply by providing the user with the ability to absorb at their own pace by exploiting the ability to pause the content [8-12]. Furthermore, podcasts can serve as a practical and valuable resource for providing a more digestible means of information such as journal articles [13-15]. Podcasts also allow the clinical community to share ideas globally and with the addition of video, they can be used for teaching procedural tasks [16-19]. As such, within the realm of anesthesia, podcasts are becoming increasingly popular as an educational tool [20].

Anesthesia podcast users report the need for a wide range of topics available as debates, journal article summaries, and mostly of short duration and multiple media [20]. The development and success of a podcast series may be influenced by the availability of content that meets the target user’s needs and inclusion of various evidence-based models for knowledge transfer and retention [21-27]. There is currently no published data on the scope of podcasts in anesthesia. Furthermore, in this growing area of e-resources for anesthesia, it is worthwhile defining and determining the factors that make for a successful podcast series. The importance of peer review and reliability of sources creating podcasts have been reported to influence their use and adoption [20,27]. There is also currently no published literature on the peer-review process for anesthesia podcasts. As such, the goals of our study were to (1) evaluate the scope of anesthesia podcasts, (2) find metrics to define success, and (3) determine factors that were associated with podcast success and podcast peer-review.

## Methods

### Ethics and Study Design

This study was exempt from ethics approval. We used a validated scoping review and content analysis approach to guide the review and characterization of available anesthesia podcasts [28]. The review was carried out on the Canadian iTunes Store. Between September 1 and September 16, 2014, we entered the keywords “anesthesia,” “anesthesia,” “anesthesiology,” “anesthesiology,” “anesthetic,” and “anesthetics” into the search field on the iTunes podcasts directory. Two reviewers (DS and CM) recorded the titles, number of episodes, and other variables (Table 1). For the eligibility assessment of the podcasts, the reviewers assessed the entire series during 2 meetings.

**Table 1:** Recorded metrics of interest for each relevant anesthesia podcast.

Category	Possible values
Authorship	Author, association of author
Country of origin	
Review process present	Yes, no
Frequency of podcast	Weekly, biweekly, monthly, and so forth
Podcast longevity	First and last episode, number of episodes
Duration	Longest and shortest episode (min)
Topic	Basic science, clinical, procedural, professional
Podcast type	Recorded didactic lecture, debate or discussion, journal summary, case presentation, practice oral exams, ground rounds, procedures
Target audience	Medical students, residents, fellows, anesthesiologists, nurse or paramedic, anesthesia assistant or nurse practitioner anesthetist
Supplemental information	Yes, no
Format	Audio, audio with PowerPoint style video, audio with real video
Availability to download	Yes, no
Presence of user feedback	Format, comments
Social media presence	Facebook, Twitter, LinkedIn, Google+

### Selection Criteria

Podcasts were initially organized as either “potentially relevant” or “not relevant” based on the title, description, and a review of the audio files. Podcasts were categorized as “potentially relevant” and included in the final analysis if they met 3 criteria: (1) One of the search terms was in the podcast description available on the store, (2) the podcast had at least one episode posted on iTunes, and (3) the podcast was in English.

Podcasts were excluded from the study if they did not have at least one episode posted on iTunes (ie, dead links) and were focused on anesthesia for veterinary services. After independent screening for relevance, the 2 reviewers met to review each podcast that had been marked as “potentially relevant” or “not relevant.” Following a literature review, we defined evidence of peer review as podcasts that were created in the context of a publication, presence of 3 or more speakers, grand rounds, and association with a journal or university [29]. For this definition, agreement was sought on each podcast title and a decision was made to include or exclude based on aforementioned criteria. Disagreements found in the review were resolved by consensus.

 

### Data Extraction and Coding

Information was extracted from the store descriptions of the apps for the variables given in Table 1. Where available, weblinks to home pages were followed to extract information verifying authorship, ability to download outside of iTunes, and the presence of supplementary resources such as notes or social media.

### Measure of Success

Although acknowledging that the success of a given podcast series should be informed in part by the ratings from the users, after pilot searches of the available anesthesia podcasts found on Canadian iTunes, it was apparent that very few of the podcasts’ series (2/22) had any user ratings or feedback. As such, we attempted to devise a mathematical model that could be used in the evaluation of podcast success based on metrics such as the length of the time the podcast series has existed, number of available episodes, and frequency of podcast publishing (further detailed later in the Results section of this paper under “Podcast Success Measure”). We proposed the use of such a model as a means of providing a measurable score of podcast success.

### Data Analysis

Descriptive analysis was used to summarize the data. Correlation coefficients with the Podcast Success Index (PSI) were determined by Pearson product-moment correlation if independent variables were continuous or by Spearman rank-order correlation if they were categorical or ordinal. Univariate generalized linear model with an identity link and normal distribution was used to identify factors associated with PSI and the evidence of a review process. Statistical significance was set at *P*<.05.

## Results

### General Podcast Characteristics, Authorship, and Affiliation

**Figure 1:**
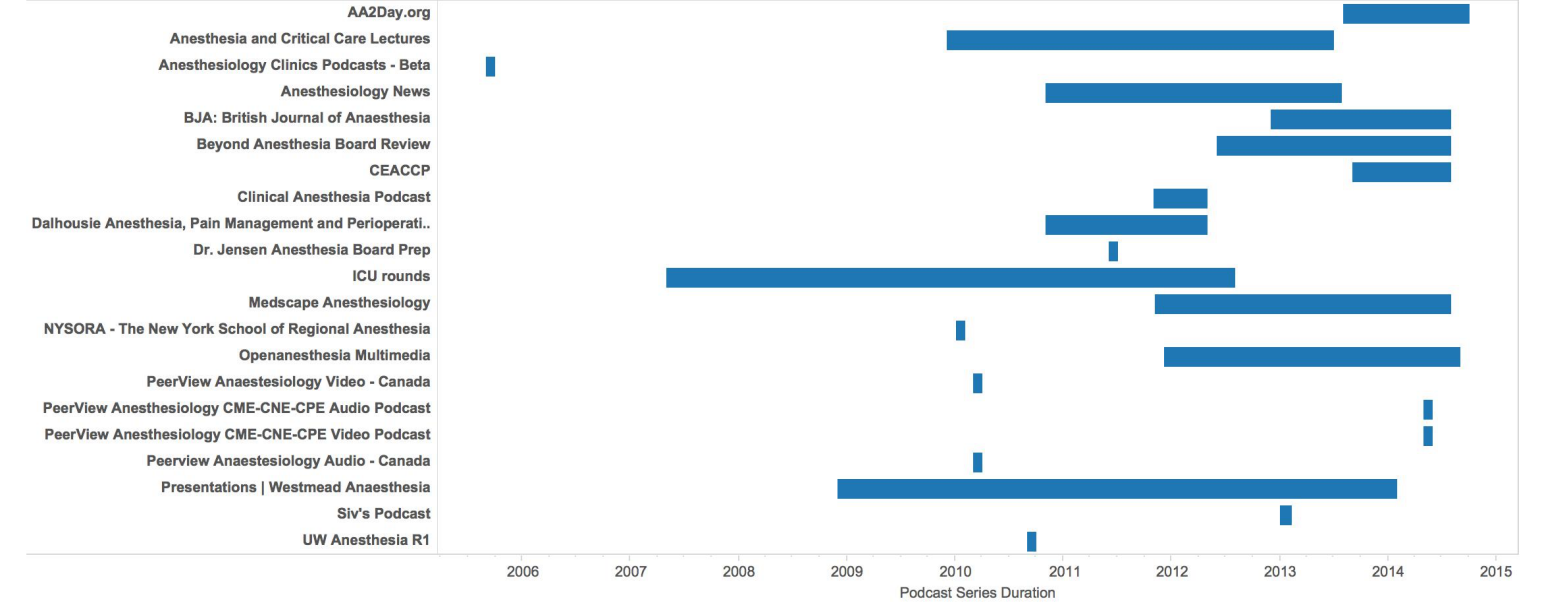
Timelines of activity for all relevant anesthesia podcasts found on iTunes Canada.

A total of 85 podcasts were found using the search terms; 63 were excluded resulting in 22 podcasts being evaluated. Most podcasts’ series 18/22 (73%) were inactive, 6/22 (27%) had not published new content in the preceding 3 months of the study (Figure 1). Most podcasts originated in the United States (15/22 = 68%) with the remainder originating in Canada, the United Kingdom, and Australia (Table 2). Less than half, 9/22 (41%), of the podcasts were produced by individuals and almost a third, 7/22 (32%), by industry. However, only a small minority, (3/22 = 14%), of the podcasts on the Canadian iTunes Store were created by anesthesia journals (Table 2).

**Table 2:** Relevant anesthesia podcast series features.

		Included podcasts, N=22 (%)
**Country of origin**		
	United States	15 (68)
	Canada	4 (18)
	United Kingdom	2 (9)
	Australia	1 (5)
**Podcast author**		
	Individual	9 (41)
	Industry	7 (32)
	Journal	3 (14)
	University	2 (9)
	Journal	1 (5)
**Podcast format**		
	Audio only	14 (63)
	Audio with PowerPoint style video	5 (23)
	Audio with real video	3 (14)
**Topics covered**		
	Clinical topics	18 (82)
	Basic science	13 (59)
	Professional	12 (54)
	Procedural	9 (41)
**Podcast types**		
	Debate	15 (68)
	Recorded didactic	6 (27)
	Journal	4 (18)
	Case presentations	3 (14)
	Grand rounds	2 (9)
	Practice oral exams	1 (5)
	Procedures	1 (5)

### Podcast Types and Length of Podcast Episodes and Podcast Series Existence Duration

**Figure 2:**
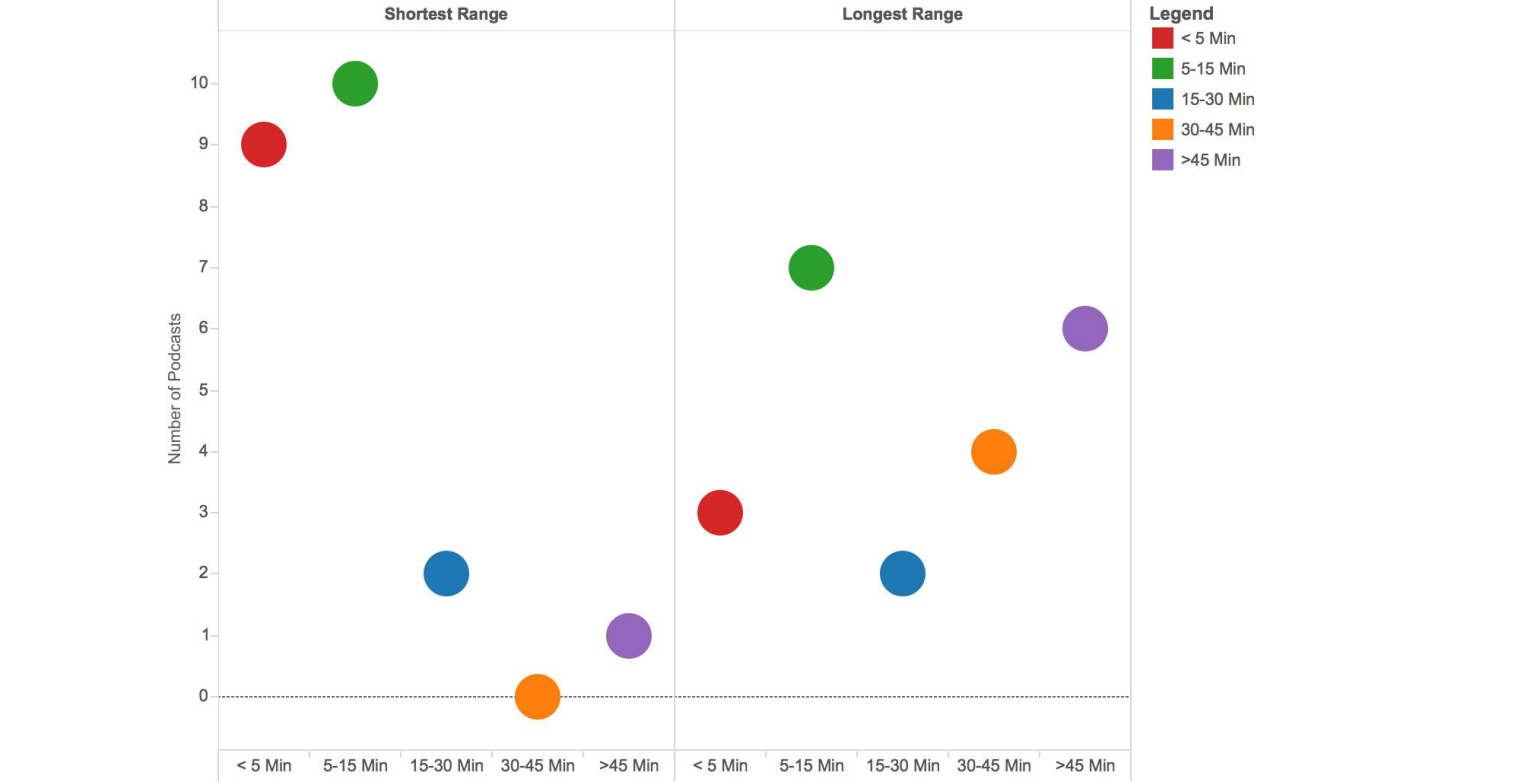
Minimum and maximum lengths of relevant anesthesia podcasts (N=22).

Podcasts ranged widely in length from less than 5 minutes to as long as 65 minutes. Eighty-six percent (19/22) of podcast series included episodes that were less than 15 minutes. Almost half of the series 10/22 (46%) also included episodes that were longer than 30 minutes (Figure 2). Over a third, 8/22 (37%), of podcasts included either video or PowerPoint slides with narration. Overall, 55% (12/22) of anesthesia podcasts were found to be downloadable outside of iTunes on dedicated websites, whereas the remainder were only available through iTunes Canada. Furthermore, 50% (11/22) of podcasts provided supplemental information in downloadable notes on dedicated websites. The median duration of existence of the podcast series was just 13 months (interquartile range, 1-39 months).

### Target Audience, Topics, Podcast Style, Peer Review

Anesthesia podcasts targeted all levels of anesthesia providers from trainees to faculty and adjunct services. Almost 80% (17/22 = 77%) of podcast series provided content directly applicable to anesthesiologists, whereas 27% (6/22) were aimed at other services such as nurses, paramedics, and anesthesia assistants.

The anesthesia podcasts covered topics that can broadly be categorized as basic science, clinical, procedural, or professionalism. Clinical topics were the most comprehensively addressed with 82% (18/22) of series covering these. Procedural topics were covered by only 41% (9/22) of podcast series (Table 2). Seventy-three percent (16/22) of podcast series demonstrated evidence of peer review. Podcasts’ series describing anesthesiologists as a target audience; that included clinical topics; and podcasts that were still actively producing content were associated with evidence of a peer-review process (Spearman *R*=0.89, *P*<.01; Spearman *R*=0.505, *P*=.02; and Spearman *R*=0.52, *P*=.01, respectively). Podcast reviews were least likely to be reviewed when created by individuals (Table 3).

**Table 3:** Univariate analysis of factors associated with a peer-review process.

Correlation	Variable	Spearman *R*	*P* value
Positive correlation			
	Podcasts targeting anesthesiologists	0.886	<.004^a^
	Podcasting with clinical topics	0.505	.02^a^
	Podcasts currently active^b^	0.516	.02^a^
Negative correlation			
	Podcasts authored by individuals	−0.505	.02^a^

^a^*P*<.05 (2-tailed).

^b^Episode in the 3 months preceding data collection.

### Podcast Style and Evaluation of Podcasts and Use of Social Media

Discussions including journal summaries were the most common podcast style (15/22 = 68%). The least used formats were practice oral exams and procedural instruction, each of which only appears in 1/22 (5%) anesthesia-related podcasts (Table 2). Most podcasts, more than three-quarters, (17/22 = 77%), were not linked to social media, whereas the remaining 5 provided links to Facebook, Twitter, Google+, and LinkedIn. The use of Twitter was associated with podcasts focusing on journal article summaries and procedural topics (Spearman *R*=0.5, *P*=.02; Spearman *R*=0.48, *P*=.03, respectively).

Only 9% (2/22) of anesthesia-related podcasts located on the Canadian iTunes store had any user feedback or rating.

### Podcast Success Measure

Ideally, to measure podcast quality and hence success, each podcast would have been assessed by descriptive and numerical user reviews. Unfortunately, this was not completed in most of the podcasts in this study. As such, in the absence of user ratings or clear peer review, we created a novel success measure, termed PSI. To address validity of the PSI, we conducted a literature search for factors that could be indicative of quality and success of podcasts. These were compiled and then distributed to podcast developers and users in both medical and nonmedical realms for review. Through an iterative fashion, a consensus was formed determining the following factors to be important in determining a successful podcast series: length of podcast existence, number of monthly episodes, ratings by users, and number of downloads/number of plays. As stated earlier, due to the lack of data on podcast user ratings, number of downloads/number of plays, we eliminated these from our equation resulting in a PSI defined by length of podcast existence and monthly frequency of publication (Equation 1 and Table 4). This PSI equation was then piloted on a random sample of nonanesthesia-related podcasts that did have user ratings and reviews to ensure correlation with PSI scores.

Podcast Success Index = log [(episodes/month) √months active] (Equation 1)

**Table 4:** Podcast success scores.

Podcast title	Date of first episode	Months active	Episodes/month	Success score
AA2day.org^a^	08/2013	14	10.29	1.59
Anesthesia and Critical Care Lectures	12/2009	44	0.27	0.26
Anesthesiology Clinics Podcasts—Beta	09/2005	1	11.00	1.04
Anesthesiology News	11/2010	38	0.92	0.75
Beyond Anesthesia Board Review^a^	06/2012	27	0.85	0.65
BJA: British Journal of Anesthesia^a^	12/2012	21	1.62	0.87
CEACCP^a^	09/2013	12	0.42	0.16
Clinical Anesthesia Podcast	11/2011	6	2.33	0.76
Dalhousie Podcast Grand Rounds—Audio	11/2010	19	0.84	0.56
Dr. Jensen Anesthesia Board Prep	06/2011	1	2.00	0.30
ICU rounds	05/2007	63	1.48	1.07
Medscape Anesthesiology Podcast^a^	01/2011	43	1.16	0.88
NYSORA—The New York School of Regional Anesthesia	01/2010	1	1.00	0
Openanesthesia Multimedia^a^	06/2009	64	3.33	1.43
PeerView Anesthesiology Audio—Canada	03/2010	1	1.00	0
PeerView Anesthesiology Video—Canada	03/2010	1	1.00	0
PeerView Anesthesiology CME/CNE/CPE Audio Podcast	05/2014	1	1.00	0
PeerView Anesthesiology CME/CNE/CPE Video Podcast	05/2014	1	1.00	0
Siv's Podcast	01/2013	1	1.00	0
The World of Anesthesiology Podcast	07/2010	34	0.88	0.71
UW Anesthesia R1	09/2010	1	3.00	0.48
Presentations|Westmead Anesthesia	12/2008	62	1.00	0.90

^a^Podcast active within 3 months of data collection (September 14).

### Factors Associated With a High Podcast Success Index

Podcasts that included fellows as the target audience demonstrated positive correlation with a high PSI (Spearman *R*=0.434; *P*=.04) (Table 5). Other podcasts targeting residents and anesthesia assistants tended toward significance. The inclusion of a wide array of topics from basic science and professional topics also demonstrated positive correlation with a high PSI. The use of Twitter was positively associated with a high PSI (Spearman *R*=0.453; *P=*.03). Interestingly, short podcasts demonstrated negative correlation with PSI (Spearman *R* = −0.506; *P*=.02).

**Table 5:** Factors associated with a high Podcast Success Index.

Characteristic	Variable	Spearman *R*	*P*
	Podcast author	0.011	.96
	Association of podcast author	0.248	.27
	Country	−0.216	.33
	Peer-reviewed	0.138	.54
	Number of ratings	0.386	.08
**Target population**			
	Med Student	0.104	.65
	Residents	0.399	.07
	Fellows	0.434	.04^a^
	Anesthesiologists	0.138	.54
	Anesthesia assistants/nurse practitioners	0.406	.06
**Podcast topics included in the series**			
	Basic science	0.456	.03^a^
	Clinical topics	0.375	.09
	Procedural topics	0.603	.003^a^
	Professional topics	0.552	.01^a^
**Podcast style**			
	Recorded didactic lectures	0.341	.12
	Debate discussion	−0.031	.89
	Journal summary	0.375	.09
	Case presentation	0.432	.04^a^
	Practice oral exams	0.017	0.94
	Grand rounds	0.227	0.31
**Other podcast factors**			
	Short podcasts (min)	−0.506	.02^a^
	Long podcasts (min)	0.388	.07
	Use of adjuncts (summary documents)	−0.036	.87
	Podcast is downloadable	0.458	.03^a^
	mp3	0.414	.06
	mp4	0.432	.04^a^
	m4v	0.201	.37
**Use of social media**			
	Twitter	0.453	.03^a^
	Facebook	0.253	.26

^a^*P*<.05 (2-tailed).

## Discussion

### Principal Findings

Our study demonstrates that anesthesia-related podcasts that have been in existence for a decade include a wide range of topics but have a high attrition rate. Using a novel podcast success tool, PSI, we have identified factors associated with podcast success: target population of podcast, type of topics covered, and the use of social media.

Our results show that podcasts in anesthesiology have been created by a wide range of authors including individuals, universities, journals, and industry. Most podcast creators have been individuals, responsible for just under half of the podcast series. Surprisingly, universities, professional organizations, and journals contribute just a small proportion of the podcasts’ series. Reasons for this may include budgetary or scope of work restrictions. Nevertheless, the journals are all recently new players in this field and more may follow suit. Although industry contributed to about a third of podcast series, industry appears to have largely exited this area as there were no active podcasts from industry during the study period. Reasons for this exit remain undetermined but may be linked to budgetary constraints and the potential presence of conflict of interest. The motivation for the creation of podcasts by individuals may include factors such as academic productivity related to education and research opportunities. Of the podcasts created by individuals, only a small minority are still active. Although studies have suggested podcasts are cheap to create and distribute, the perceived lack of quality content is a known major factor limiting wider adoption [20,27]. Our study does demonstrate that the podcast series duration for many podcasts was just a median of 13 months (interquartile range, 1-39 months). This is akin to a television show that lasts only one season and does not get renewed for subsequent seasons. Other reasons for this rather short existence of podcast series may be explained by the challenges of producing high-quality podcasts. These have been reported to be good quality content and cost related to the state of the art audio production equipment, associated personnel, time, and the presence of submatter experts [20]. These factors may contribute to the low number of individuals creating anesthesia-related podcasts. Current and new podcasts creators will need to consider these issues and challenges to ensure their podcasts’ series run as long as “Sesame Street.”

A major goal of this work was to develop a mathematical model that could assess the success of the podcasts using data that are currently available. After reviewing the collected data and metrics available for podcast series in anesthesia, it became apparent that a key element was missing to assess quality and impact: user feedback. Only 9% of podcasts had any review on iTunes. This may be explained in part by the structure and function of iTunes, which does not make it easy to evaluate podcasts. Nevertheless, using available data and metric, we developed the “PSI” formula weighted toward podcast productivity and longevity. A podcast author who provided frequent episodes over an extended period could be said to be more successful than a less productive or less long-lasting counterpart; much the way one could evaluate the popularity of a periodical. The use of such an index may assist users with filtering the quality of podcasts and assessing for relevance. It must also be stated that this is a quantitative rating. Recently, podcast assessment rubrics have been proposed consisting of qualitative evaluation criteria that could be used in conjunction with the PSI to enhance assessment of podcast quality and success [30].

It is important to point out that user feedback may improve the utility of our PSI by a user informed dimension of quality. In our data, there was minimal social media presence among the included anesthesia podcasts limiting the inclusion of social media user generated reviews. In contrast, Thoma et al looked at a Social Media Index, proposing the incorporation of social media “likes” and “follows” as well as page ranks of the resource as a quality assessment model of websites and e-resources in emergency medicine [29]. The use of e-resources in emergency medicine is more widespread allowing the existence of many users who provide numerous reviews and feedback on various platforms including social media. Anesthesiology may still be in infancy with regard to the use of e-resources and not have as highly interactive user body.

Nevertheless, the association of factors such as including a wide range of topics in a podcast series with a high PSI suggests that the podcasts may be meeting needs in a broad target population. Creators of podcasts should continue to develop series that provide relevant and pertinent information from broad topics. Short podcasts and case discussions were associated with a higher PSI and were consistent with those from previous work surveying podcast preferences of Canadian anesthesia residents [20].

The high rate of evidence of peer review (73% of podcast series) was an unexpected finding. This may be due to our definition for evidence of peer review, which may have been liberal. The association of a peer-review process with podcasts targeting anesthesiologists suggests that users may regard podcasts as providing some level of reliable and valid information. However, it will be important for podcast creators to publish their review processes to better inform end users on the reliability and relevance of these resources. More importantly, podcast series created by individuals were least likely to be reviewed. The inclusion of a review process may be a logistic challenge for such individual publishers of podcasts. It is important users of these podcasts take time to familiarize themselves with the producers and the content.

Our study provides new data on the scope of and success of podcasting in anesthesia albeit with some limitations. The first is that our podcasts were limited to the Canadian iTunes Store, which will not show the results of content exclusively available in other countries or regions. This may have contributed to our limited sample size. However, iTunes works as a geofence and so our study sample is relevant to all those who access podcasts in geographical Canada. Furthermore, the majority of the podcasts were from the United States. However, further work could extend the survey to a global level with the inclusion of both international iTunes stores and other pod catcher platforms such as soundcloud, archive.org, and Podomatic. In addition, in terms of individual podcast topics, we assessed broad topic categories such as clinical, procedural, professional, and basic science. The previous work by Matava et al surveyed current residents regarding desired topics was more robust, delving into subcategories of the broader classifications [20]. This analysis could be addressed in future works but would require closer analysis of each and every podcast episode that was not deemed appropriate for our study.

### Conclusion

This study is the first to provide a scoping review, critical analysis of the success of the anesthesiology e-resource—podcasts. We demonstrate that podcasts' series for anesthesiology cover a broad area of topics but are relatively short-lived. Anesthesia podcasts demonstrate high-level peer-review processes in podcasts. Factors such as including particular target populations, type of topics covered, and the use of social media correlate with podcast series success, as defined by a novel PSI. The continued growth in this area may depend on further work involving social media integration and continued inclusion of wide range of topics.

## Abbreviations

PSI: podcast success index
